# Supplementary material for: Snacking Behaviours of Australian Young Adults: Secondary Analysis of the MYMeals Cross-Sectional Study
Source: Nutrients. 2023 Oct 22;15(20):4471. doi: 10.3390/nu15204471 (PMC10609995; doi:10.3390/nu15204471)
Supplement: Supplementary file 1 [file nutrients-15-04471-s001.zip › nutrients-2642987-supplementary.pdf]

Supplementary Table S1. Snack consumer demographic characteristics for consumers of snacks for the total sample and for those classified as acceptable energy reporters**.**

**Participant characteristics**		**Total sample n = 889 (%)**	**Acceptable energy reporter[b] n = 589 (%)**
Total sample		889	589
Gender	Female	520 (58)	354 (60)
	Male	369 (42)	235 (40)
Age group (years)	18-24	479 (54)	328 (56)
	25-30	410 (46)	261 (44)
Body Mass Index (kg/m^2)	Underweight (<18.5)	47 (5)	41 (7)
	Healthy weight (18.5-24.99)	501 (56)	372 (63)
	Overweight (25-29.99)	224 (25)	129 (22)
	Obese (>29.99)	117 (13)	47 (8)
Socioeconomic status [a]	Low	363 (41)	229 (39)
	High	526 (59)	360 (61)

[a] From Socio-Economic Indexes for Areas Index for Relative Advantage and Disadvantage [18] based on residential postcode, lowest five deciles = low, highest five deciles = high. [b] Cut-off for Energy Intake: Basal Metabolic Rate (BMR) for an individual based on three days, low energy reporters < 1.0 x BMR, high energy reporters > 2.4 x BMR were excluded [22].

Supplementary Table S2. Median (IQR) contribution (%) of energy, protein, total sugars, sodium, and saturated fat from snacking to total energy and nutrient intake in participants (n=589) who consumed at least one snack during three days recording period and were acceptable energy reporters.

	**Sample size, n**	**Energy**		**Protein**		**Total sugars**		**Sodium**		**Saturated fat**	
		Median (%)	IQR	Median (%)	IQR	Median (%)	IQR	Median (%)	IQR	Median (%)	IQR
**Acceptable energy reporters, consumers**	589	13.9	13.7	6.8	8.8	24.3	24.5	8.6	12.3	18.1	21.1
**Age (years)**											
18-24	328	13.9	13.3	6.5	8.4	23.9	24.6	8.2	11.2	18.5	20.5
25-30	261	14.1	15.1	7.3	9.5	24.9	24.8	9.0	13.2	18.1	21.9
**p-value**		0.367		0.206		0.79		0.251		0.649	
**Gender**											
Male	235	12.9	13.2	6.4	9.3	20.7	20.9	8.0	13.3	16.5	17.8
Female	354	14.8	14.1	7.2	8.4	26.9	26.1	8.9	11.8	19.6	23.8
**p-value**		**0.005**		0.06		**0.002**		0.145		**0.016**	
**SES**											
Low	229	14.3	14.7	7.3	9.0	23.9	24.1	9.3	12.7	18.4	21.9
High	360	13.7	13.5	6.8	8.8	24.7	24.3	8.1	11.6	17.8	20.2
**p-value**		0.74		0.72		0.322		0.256		0.858	
**BMI**											
Underweight (<18.5)	41	12.9	12.4	5.7	8.0	25.9	22.6	8.5	12.3	15.0	18.5
Healthy Weight (18.5-24.99)	372	14.3	14.1	6.8	9.2	24.2	25.1	8.1	12.1	18.1	22.4
Overweight (25-29.99)	129	13.2	15.2	7.6	8.9	24.1	23.9	9.3	13.1	18.4	19.3
Obese (>29.99)	47	15.0	14.1	6.9	8.5	24.3	27.4	9.3	13.1	18.9	22.5
**p-value**		0.841		0.820		0.982		0.812		0.555	

Supplementary Table S3. Median (IQR) of average total energy, protein, total sugars, sodium, and saturated fat intake across three days from snacking in participants (n=589) who consumed at least one snack during the three-day recording period and were acceptable reporters.

Characteristics	Sample size, n	Total energy (kJ)		Total protein (g)		Total sugars (g)		Total sodium (mg)		Total saturated fat (g)	
		Median	IQR	Median	IQR	Median	IQR	Median	IQR	Median	IQR
**Acceptable energy reporters, consumers (total)**	589	1214	1218	6.1	8.3	18.9	21.8	222.0	323.3	5.3	7.0
**Age (years)**											
18-24	328	1171	1213	5.8	7.7	19.0	20.1	205.5	304.0	5.2	7.0
25-30	261	1301	1317	6.8	8.9	18.9	23.9	236.4	333.2	5.4	7.9
**Gender**											
Male	235	1279	1479	7.0	10.6	19.0	21.8	243.9	433.0	5.7	6.8
Female	354	1177	1171	5.8	7.1	18.6	21.9	210.3	279.1	5.0	7.1
**SES**											
Low	229	1298	1185	6.5	7.9	20.2	21.4	251.6	325.3	5.3	7.2
High	360	1195	1235	5.8	8.6	18.1	22.0	209.2	310.7	5.3	6.9
**BMI**											
Underweight	41	1043	967	4.7	6.3	15.4	21.5	177.8	222.7	4.4	4.4
Healthy weight	372	1177	1210	6.0	8.2	18.3	21.1	210.3	296.0	5.0	6.6
Overweight	129	1344	1337	6.9	9.1	18.9	21.3	263.3	403.7	6.2	8.0
Obese	47	1557	1885	7.1	8.7	23.1	33.4	272.0	380.	7.7	10.0
